# Prospects for clinical use of reprogrammed cells for autologous treatment of macular degeneration

**DOI:** 10.1186/s13069-015-0026-9

**Published:** 2015-05-15

**Authors:** Ana Belen Alvarez Palomo, Samuel McLenachan, Fred K Chen, Lyndon Da Cruz, Rodney J Dilley, Jordi Requena, Michaela Lucas, Andrew Lucas, Micha Drukker, Michael J Edel

**Affiliations:** Control of Pluripotency Laboratory, Department of Physiological Sciences I, Faculty of Medicine, University of Barcelona, Hospital Clinic, Casanova 143, 08036 Barcelona, Spain; Centre for Ophthalmology and Visual Science (Lions Eye Institute), University of Western Australia, 2 Verdun Street, Nedlands, WA 6009 Australia; Moorfields Eye Hospital, 162 City Road, London, EC1V 2PD England; Ear Sciences Centre, 1 Salvado Rd, Subiaco, WA 6008 Australia; School of Surgery, University of Western Australia, 35 Stirling Highway, Nedlands, WA 6009 Australia; School of Medicine and Pharmacology, University of Western Australia, 35 Stirling Highway, Nedlands, WA 6009 Australia; PathWest, SCGH Laboratories Hospital Ave, Nedlands, WA 6009 Australia; Institute for Immunology and Infectious Diseases, Murdoch University, Building 390, Discovery Way, Murdoch, Perth, WA 6150 Australia; Helmholtz Zentrum München, German Research Centre for Environmental Health (GmbH), Institute of Stem Cell Research, Ingolstädter Landstraße 1, D-85764 Neuherberg, Germany; Division of Pediatrics and Child Health, Westmead Children’s Hospital, Corner Hawkesbury Road and Hainsworth Street, Westmead, Sydney, NSW 2145 Australia; School of Anatomy, Physiology & Human Biology and Centre for Cell Therapy and Regenerative Medicine (CCTRM), University of Western Australia, 35 Stirling Highway, Nedlands, WA 6009 Australia

## Abstract

Since the discovery of induced pluripotent stem cells (iPSC) in 2006, the symptoms of many human diseases have been reversed in animal models with iPSC therapy, setting the stage for future clinical development. From the animal data it is clear that iPSC are rapidly becoming the lead cell type for cell replacement therapy and for the newly developing field of iPSC-derived body organ transplantation. The first human pathology that might be treated in the near future with iPSC is age-related macular degeneration (AMD), which has recently passed the criteria set down by regulators for phase I clinical trials with allogeneic human embryonic stem cell-derived cell transplantation in humans. Given that iPSC are currently in clinical trial in Japan (RIKEN) to treat AMD, the establishment of a set of international criteria to make clinical-grade iPSC and their differentiated progeny is the next step in order to prepare for future autologous cell therapy clinical trials. Armed with clinical-grade iPSC, we can then specifically test for their threat of cancer, for proper and efficient differentiation to the correct cell type to treat human disease and then to determine their immunogenicity. Such a rigorous approach sets a far more relevant paradigm for their intended future use than non-clinical-grade iPSC. This review focuses on the latest developments regarding the first possible use of iPSC-derived retinal pigment epithelial cells in treating human disease, covers data gathered on animal models to date and methods to make clinical-grade iPSC, suggests techniques to ensure quality control and discusses possible clinical immune responses.

## Review

### iPSC to treat animal models of human disease

Many human diseases have been successfully ‘treated’ in animal models with induced pluripotent stem cells (iPSC) or human embryonic stem cell (hESC)-derived cells, such as sickle cell anaemia [1] (blood cell replacement), amyotrophic lateral sclerosis (ALS) [2-4] (motor neuron cell replacement), age-related macular degeneration (AMD) [5-7] (retinal pigment epithelial (RPE) cells), spinal cord injury [8-10] (neural stem cells) and Parkinson’s disease [11-13] (dopaminergic neuron replacement), providing essential *in vivo* pre-clinical data. A great level of confidence can be drawn from the animal data to provide a solid platform to move towards clinical trials in the near future. This review focuses on the latest developments regarding the first possible use of iPSC-derived retinal pigment epithelial cells in treating human macular degeneration.

### Rat model for macular degeneration

Many different animal models have been used to test RPE cell function *in vivo* [14,15]. The Royal College of Surgeons (RCS) rat is an animal model of retinal pigment epithelium dystrophy that has been used extensively to demonstrate the proof of principle and mechanism of visual rescue in RPE transplantation. This dystrophic strain of rat has a recessive mutation in the *MerTK* gene that results in failure of RPE cells to phagocytose rod outer segments [16-18]. The consequences of this mutation include accumulation of subretinal debris, death of rod photoreceptor cells and later cone cells, secondary inner retinal degeneration, retinal vascular changes and central adaptive modulation in neural circuitry.

Retinal degeneration in the RCS rat can be prevented or reversed by subretinal transplantation of RPE cells from a non-dystrophic congenic strain [19,20]. The RCS rat has been used by a number of groups to examine different potential cell sources for RPE replacement for the treatment of AMD and other retinal diseases (reviewed by Carr et al. 2013) [21], including human foetal and neonatal RPE [22,23], adult human RPE cell lines, such as ARPE19 [24-26], stem cell-derived RPE from hESC [5,27,28] and iPSC [6]. In all of these studies, transplanted RPE cells resulted in the preservation of the photoreceptor layer, demonstrating the feasibility of treating RPE dystrophy with cell therapy.

Interestingly, transplantation of neurospheres derived from the human foetal forebrain into the RCS rat retina was shown to rescue retinal degeneration in the absence of RPE cell differentiation. Transplanted human neural stem cells were shown to persist in the host for up to 4 months after transplantation and exhibited phagocytic activity, resulting in the clearance of subretinal debris and preservation of photoreceptor cells and retinal histology [29]. These observations suggest that neural stem cells provide neurotrophic support to the retina; however, the uptake of subretinal debris by transplanted cells suggests that additional functions such as phagocytic clearance may be engaged in transplanted cells.

Transplantation of healthy RPE into the submacular space has shown promising potential as a cure for AMD and could offer an alternative to regular intraocular injections of vascular endothelial growth factor (VEGF) blocking drugs. Current surgical approaches to reconstruct RPE in wet and dry AMD include full macular translocation [30,31], which involves repositioning the retina over a new patch of healthy RPE, and autologous RPE-choroid patch grafting [32], which involves harvesting healthy RPE from peripheral retina. Short- and long-term outcomes of translocation surgery in wet AMD have provided proof of principal that visual function can be rescued by RPE transplantation [33,34]. However, there are significant limitations in these autologous techniques related to surgical complications [33]. Given these limitations, considerable effort has been invested in the development of a simplified surgical approach that utilises a pre-made RPE patch for retinal transplantation.

### Methods to make clinical-grade iPSC

The generation of clinical-grade human iPSC (hiPSC) should take into consideration several aspects of safety and applicability, namely genome integrity, avoiding the use of oncogenes, the use of xeno-free materials and good manufacturing practices, high efficiency and practical considerations to create a widely used cell therapy approach.

### Reprogramming without leaving traces in the genome

Since Yamanaka’s method to make iPSC, using retrovirus for Oct4, Sox2, Klf4 and c-Myc genes, a reprogramming method that does not leave permanent modifications of the genome has been sought [35]. Different groups have developed protocols to induce iPSC lines using excisable lentiviral [36], transposon [37] or non-integrating episomal vectors [38].

To avoid any remote risk of changes in the genome, DNA-free methods have also been designed. Warren and colleagues [39] were the first to show the induction of iPSC from primary human fibroblast cultures by using *in vitro* transcribed mRNA of the four classic reprogramming factors described by Yamanaka. Other groups have also achieved reprogramming by methods such as direct transfection of recombinant cell-penetrating reprogramming proteins in primary mouse embryo fibroblasts [40] or a combination of chemicals [41]. Methods do exist to use xeno-free, virus-free, c-Myc-free and feeder-free methods to make iPSC [42,43]. We are adapting published methods [39,44,45] to develop a new protocol for clinical-grade iPSC from human cord blood stem cells and xeno-free human fibroblasts. The next challenge will be to establish internationally accepted guidelines to determine the quality of iPSC.

### Xeno-free and good manufacturing procedures

To create iPSC lines and derivatives that are acceptable for clinical use, it is necessary to use reagents for cell culture, surface attachment and cell passage that are chemically defined and free of any contamination of chemicals that cause an immune response. Feeder-free cell culture systems have been generated that can be adopted for clinical use [46]. In these conditions, cell reprogramming is accomplished without a feeder layer by plating cells on vitronectin, allowing for controlled and reproducible conditions. Defined xeno-free media as well as attachment and passaging reagents are now commercially available from different manufacturers.

Another important aspect in the creation of clinical-grade iPSC is to identify and follow good manufacturing practices (GMP) for all processing steps. The production of iPSC from skin biopsies entirely following GMP protocols have been generated following standard operating procedures (SOP) [47] for each of the methods involved in the generation and characterisation of iPSC lines [48]. They have classified SOP in five broad categories: administrative, material management, equipment, manufacturing and quality control and assurance. However, to date, SOP have only been described for retrovirally generated iPSC, and specific SOPs will need to be made for non-virally generated methods. Quality control standards for the clinical use of iPSC are still to be defined, and they will have to assess aspects such as genetic stability, safety, purity and identity.

### High efficiency

High efficiency is a critical feature of any reprogramming protocol that is suitable for the creation of clinical-grade iPSC from patient cells. The addition of chemical inhibitors of, for example, the p38 pathway, inositol trisphosphate 3-kinase, Aurora A kinase and TGF-β receptor, has been shown to increase significantly the efficiency of the reprogramming process [49]. Also, it has been shown that depletion of nuclear protein mbd3 unblocks Oct4 and Sox2 promoters for activation, leading not only to a dramatic increase in efficiency but also to a considerable reduction in the reprogramming time compared to standard reprogramming protocols [50].

### Leaving out the oncogenes

One critical aspect in reprogramming cells to pluripotency is the increased oncogenic potential of cells as a result of genetic manipulation. This is of special concern when introducing oncogenes such as Klf4 and particularly c-Myc. Although it is possible to use reprogramming cocktails without c-Myc, the efficiency is dramatically decreased [51]. In many cell types, c-Myc is only transiently expressed in reprogramming protocols and is a powerful oncogene able to induce cancer and gene amplification [51,52]. We are trying to dissect cell cycle effects of c-Myc, necessary for the reprogramming process, from the undesirable tumorigenic effects by investigating the use of other cell cycle-related genes with the aim of identifying a safe substitute for c-Myc [43]. Other genes, such as the translation enhancer lin28 or chromatin modifying factors, have been used as an alternative approach to substitute potential oncogenes when reprogramming [53].

### iPSC bank from compatible donors

One of the attractive features of iPSC technology is the possibility of creating autologous cell therapies using a patient’s adult cells as a donor source, avoiding the immunological reactions likely to grafted non-autologous cells. However, the personalised production of iPSC for cell therapy would certainly be a costly and lengthy approach. The use of iPSC from immunologically compatible donors would allow the creation of cell banks with ready-to-use iPSC and derivatives that could be transplanted in a short time and with minimal rejection [54]. It has been estimated that ten cell lines homozygous for common HLA antigens (HLA-A, HLA-B, HLA-DR) would match 37% of the UK population and be beneficial to 67% [55]. Efforts in Europe are currently being made to bring together national blood banks to collect enough homozygous cord blood units to match a significant percentage of the population.

### Methods to evaluate the quality of iPSC

The question of iPSC quality depends on the classification or definition of what we mean by the pluripotent state of cells. Currently, we are aware of at least two states for pluripotent stem cells, naive and primed [56], and often cultures of ESC and iPSC exhibit characteristics of both [57]. Some culture conditions may even promote reversion of iPSC to a totipotent state [58]. The criteria for defining the respective stages of pluripotency could include:

1. X chromosome inactivation state - female naive cells harbour two active X chromosomes, while primed cells possess one inactive X. This can be assayed by analysing XIST transcription, a non-coding RNA that initiates coding of one copy of the X chromosome leading to its inactivation in primed cells [56], and by analysing lysine 27 tri-methylation modification of histone 3 (H3K27me3) on the X chromosome [59].
2. Bivalent domain occupancy consisting of H3K27me3 and H3K4me3 modifications in the promoters and bodies of developmental genes, respectively, is more abundant in primed cells relative to naive cells [60]. This has been proposed to contribute to transcriptional induction of developmental genes when iPSC are promoted to differentiate [61].
3. Activation of specific signalling cascades - TGF-β in response to activin and receptor tyrosine kinase in response to FGF in primed miPSC and hiPSC [62] and inactivation of Erk signalling (or bypass stimulation by LIF [63]) in naive miPSC and hiPSC [56]. Typical cultures of hiPSC are thought to exhibit characteristics of the primed state.

These parameters potentially lay the foundation for the testing of pluripotency of iPSC clones, typically following ten passages. To qualify as undifferentiated hiPSC, clones are typically analysed to meet the following criteria:

1. Self-renewal as compact flat colonies in the presence of media supplemented with basic FGF and activin A (or TGF-β).
2. Expression of core pluripotency factors, including (but not limited to) OCT4, SOX2, NANOG (and LIN28 on occasions) on the transcriptional and protein levels, and alkaline phosphatase activity.
3. High histone bivalent domain occupancy (tested infrequently) [57].

Clones that exhibit properties of undifferentiated hiPSC are then further tested to analyse their ability to give rise to progeny of the three germ layers. Classical tests include differentiation tests *in vitro* and *in vivo* [64]:

1. Typical *in vitro*-based assays rely on treatment of clones by media containing growth factors that promote cell specification, for example, activin A, BMP4 and Wnt3a, in the presence or absence of FBS without basic FGF. Within 4 to 6 days, the cells are tested for gene and protein expression in embryonic lineages, including the classical markers Pax6, Brachyury and Sox17 (ectoderm, mesoderm and endoderm embryonic tissues, respectively). Depending on the concentrations of the factors and analysis time points, differentiated hiPSC should present these markers [65].
2. The teratoma assay tests the capacity of hundreds of thousands of transplanted cells to give rise to teratoma tumours in immunodeficient mice. Many host locations have been tested, among which the most popular are subcutaneous injection and kidney capsule engraftment. Clones that give rise to benign tumours consisting of cells from the three germ layers, represented typically by neural rosettes, cartilage and gastrointestinal-like epithelium (ectoderm, mesoderm and endoderm, respectively), are considered pluripotent [64].

Assessing genetic aberrations is an additional important factor for determining the quality of hiPSC. Such aberrations can result from cellular stress that accompanies reprogramming [66], selection during clone propagation and from aberrations in the original somatic cells. Characterisation assays typically rely on analysis of gross chromosome (cytogenetic) integrity. Giemsa staining is an inexpensive technique that provides resolution of several megabases. Considerably more expensive techniques are fluorescent *in situ* hybridization (FISH)-based and comparative genomic hybridization (CGH) assays that can identify abnormalities up to the level of individual genes. Genomic approaches that are based on microarrays and next-generation sequencing (NGS) are also becoming popular for genomic characterisation of hiPSC, and it is expected that, as prices go down and analysis tools improve, they will become the standard, replacing the cytogenetic tests [67]. However, the introduction of genomic approaches makes it easier to detect mutations and consequently it becomes more difficult to decide on the definitions of hiPSC quality. Clones bearing aberrations spanning thousands of base pairs are generally regarded as poor quality, but quality assessment of clones bearing several mutations is much more complicated since it is difficult in many cases to assess the influence of subtle mutations on cellular behaviour and, similarly, it is difficult to assess the functional relevance of SNPs [68]. Importantly, current genomic approaches cannot replace cytogenetic tests since the latter provide important information concerning the proportion of aberrant cells, while the genomic approaches are based on cell averages. Therefore, quality control assessment of hiPSC should first include a combination of cytogenetic and genomic assays.

We expect in the future that assessing hiPSC quality will incorporate assays that are based on single-cell genomics and posttranscriptional modifications of pluripotency genes and on methods that combine both. This is because it is has been shown that iPSC exhibit unique signatures of posttranscriptional modifications of mRNA, especially at the 3′ UTR [69,70], and also because single-cell genomics will provide information concerning the proportion of normal/genetically aberrant cells [71]. In addition, we expect that epigenetic analysis will become common for evaluating hiPSC quality since it is likely that some adult tissue DNA methylation markers, and possibly histone markers, are maintained in iPSC [72].

### Clinical application of iPSC

The future use of iPSC in the clinic largely relies on data collected from past and current clinical trials using hESC-derived cells [73,74].

### iPSC to treat macular degeneration in humans

For hESC-RPE cells, there are currently two cell products in clinical trials: Advanced Cell Technology (ACT) - MA09-hRPE cells and Pfizer-UCL clinical trial - PF-05206388 Living Tissue Equivalent. To date, Dr Masayo Takahashi (Laboratory for Retinal Regeneration, RIKEN Center for Developmental Biology) regulatory approval has been in place since 2014 to conduct the first phase I clinical trial using hiPSC-RPE in collaboration with the Institute for Biomedical Research and Innovation and with support from the Kobe City Medical Center General Hospital.

The first human stem cell transplantation trial by ACT used immunosuppression, and this is also planned for the Pfizer London project trial. Stem cell sources for retinal repair have been identified and validated including human embryonic stem (hES) cells [5,28] (ACT and Pfizer-UCL), induced pluripotent stem cells [75,76] (RIKEN Center for Developmental Biology), retinal tissue [77], umbilical tissue stem cells and retinal progenitor cells [78,79]. Therefore, many groups have demonstrated successful conversion of stem cells to human RPE or neuro-retinal cells, particularly photoreceptors.

One of the primary outcomes of the hESC-RPE cell suspension trial is ‘any evidence that the cells showing tumorigenic potential’. Animal pre-clinical data showed lack of tumour formation in animals receiving 50,000 to 100,000 RPE cells spiked with 1% undifferentiated hES cells, and there was no tumour development after 4 months in human subjects who received the hESC-RPE which had less than 1% residual undifferentiated hES cells. It is reassuring that the potentially undifferentiated hES cells injected into the subretinal space have not migrated elsewhere in the eye, or systemically, causing tumour formation. It is not certain whether the transplanted hESC-RPE survived the procedure in these patients since there were no data from *in vivo* imaging of individual RPE cells.

An alternative to allogeneic sources of pre-made RPE is patient-specific RPE derived from somatic cells, either directly using defined RPE factors or via iPSC [80]. However, there are no data on the benefit of transplanting these cells in animal models of retinal degeneration. Autologous induced pluripotent stem cells have been seen as advantageous as a source for retinal regeneration because of the theoretical benefit of not needing immunosuppression and the ethical attraction of using adult rather than embryonic tissue. In the RIKEN iPSC-RPE trial, patients with wet AMD are recruited, but there is a 10-month lag time between skin biopsy to harvest autologous cell source and shipment of iPSC-RPE cell sheets for transplantation. However, there is little published information of the technique of iPSC derivation and RPE characterisation for individual patients. The estimated 10-month lag time and the USD 100,000 price tag for manufacturing the iPSC-RPE patch make this technique more expensive than the alternative and effective treatment in wet AMD such as ranibizumab or aflibercept therapy.

Despite the enthusiasm for stem cell therapy, there remain a number of unaddressed, fundamental problems in using allogeneic cells as a donor source in cell therapy for AMD. The most important is immunological rejection. It is notable that the hES-RPE cell therapies being pursued by pharmaceutical companies are based on single hES cell lines with a propensity for spontaneous RPE differentiation. However, such allogeneic hESC lines necessitate the use of immunosuppressive drugs to allow the survival of foreign cells in the eye. It is also notable that all previous attempts to correct AMD using allogeneic RPE cells (such as foetal or cadaveric RPE) failed due to tissue rejection [81].

Although iPSC-derived RPE cell transplantation clinical trials are underway in Japan, the iPSC were produced using non-clinical methods by retroviral or Sendai virus induction and included the oncogene c-Myc in the reprogramming factor cocktail [82]. While these methods are useful for producing iPSC for research purposes, we do not consider them appropriate for clinical translation. The development of clinical-grade iPSC for therapeutic applications such as the treatment of AMD is an essential step that remains to be integrated into iPSC-based cell therapy protocols and to receive appropriate regulatory approval.

### Regulation of clinical trials

In any new area of translational science and potential therapy, such as stem cell treatments, the initial steps into human treatment are often the longest and hardest. Part of the reason is that there is no framework or experience in terms of guiding research direction. Scientists, clinicians and regulators alike are entering new and unfamiliar areas and, as such, progress appears slow. Regulation of hESC-derived stem cell trials have been centred on safety and tumorigenicity studies of the donor and derived cells. The advantage of this approach is that it is finite with a single source of cells and a potentially repeatable method to produce RPE. Issues such as GMP standards for RPE cells, clinical trial planning, critical areas of collaboration and clinical trial design have recently been reviewed [83].

The UCL-Pfizer partnership undertaking the development of hESC-RPE uses a polyester substrate carrier to maintain polarity and differentiation of grafted cells. The approval process for this cell-patch combination is more complex than hESC-RPE cell suspension grafts since the substrate, matrix coating and surgical delivery device all require individual regulatory approval in addition to the hESC-RPE cell product. Another major difference between the cell-patch trial and the RIKEN unsupported RPE sheets trial is the patient inclusion criteria - the UCL-Pfizer study will enrol patients with wet AMD rather than dry AMD. The purpose is to restore vision by removing choroidal neovascularisation and restoring submacular RPE to allow visual recovery, as opposed to preventing further loss of vision in dry AMD, as is the case for anti-VEGF treatments. Since the natural history of untreated wet AMD is a rapid decline in vision, the clinical effect will be apparent within 6 months, whereas in the dry AMD trial, it may be many years or decades before clinical benefit is apparent because of slow disease progression. In both hESC-RPE trials, immuno-suppression is required due to the theoretical risk of rejection.

Induced pluripotent cells from autologous donors could present a regulatory challenge as each treatment will be from a different donor and therefore represent a different cell line. It will not be feasible to regulate each cell line as a new entity due to time and resource reasons, so there will need to be a dialogue between researchers and regulators to resolve this. Initially, as more information comes from the early hESC trials (ACT and Pfizer-UCL) and retinal iPSC trial (RIKEN Center) there will be a better idea of safety, stability of the cells and data that can be used to inform future regulation.

### Clinical immunology of iPSC

The potential of iPSC to assist the regeneration, supplementation or replacement of human tissues is currently being realised through the evaluation of differentiated tissues in small-animal models. For human clinical applications to be developed from this point, the following immunological issues must be addressed.

Firstly, potential overexpression of proteins that are not typically expressed in the target differentiated tissue is a concern as this might introduce neo-antigens to which the host might mount an immune response. Such a scenario was reported by Zhao and colleagues [84] who described the increased expression of a range of gene products in teratomas derived from either lentiviral vector- or episomal-driven iPSC of syngeneic murine embryonic fibroblasts which were implanted subcutaneously. They further demonstrated that ectopic expression of some of these genes (*Hormad1* and *Zyg16*) in ESC lines could induce immunogenicity as indicated by their failure to form teratomas after implantation in immunocompetent mice, with this immunity dependent on both CD4+ and CD8+ T-cells. Of note, *Hormad1* expression has been reported as a tumour antigen [85]. Two recent studies have separately tested the immunogenicity of iPSC-derived cells using quite different approaches. Araki et al. produced iPSC using lentiviral vectors from either murine embryonic fibroblasts and used these cells to create highly chimeric syngeneic mice. Tissues such as the skin or bone marrow from these chimeric mice were then grafted into syngeneic hosts with no indications of immunity against the grafts [86]. Guha et al., using tail tip fibroblasts, produced tissues derived from three different embryonic germ layers and implanted them under the kidney capsule. Again, no evidence of graft-associated immunity could be detected [87]. Interestingly, the study reported that embryoid bodies (EB) derived from ESC, iPSC lines and the episomal iPSC line, Epl, previously used by Zhao et al., all expressed high levels of *Hormad1*, *Zg16* and *Retn*, whilst differentiated cells derived from each of these lines did not.

Additional support for the successful use of iPSC as a cell therapy has been shown in a study by Morizane and colleagues where neuronal cells derived from human leukocyte antigen (HLA)-matched non-human primate iPSC, generated by both lentiviral and non-integrating episomal methods, were successfully engrafted by injection into the brain of host monkeys, in the absence of immunosuppression, and tolerated for greater than 3 months without signs of infiltration by MHC II+ host microglia or CD3+ T-cells. Interestingly, neuronal grafts derived from HLA-mismatched donor cells, although infiltrated by microglia and CD3+ T-cells, also survived beyond 3 months but at a lower number and density [88]. Such recent results should provide encouragement for additional studies using a wider range of iPSC differentiated cell types in both murine and non-human primates and ultimately in clinical studies.

Secondly, the length of time necessary to achieve the production of graft materials may be too long to be practical, an issue that is directly related to the efficiency of the iPSC process utilised. The methodology to generate iPSC is still under rapid and continual development, with alternate delivery systems being evaluated, each having potentially different immunogenic responses [39,89]. We have recently reported on the development of a novel culture environment, which includes the addition of plant hormones such as auxin and cytokinin to the retroviral infection cultures which resulted in a twofold increase in the efficiency of iPSC development and, in addition, showed the effect of downregulation of the c-Myc oncogene [90]. Thus, we demonstrated that plant hormones might have a role in increasing the efficacy and safety of iPSC for cell replacement therapy. It is critical to translate these recent developments to the production of hiPSC so that they can be carefully characterised with respect to that of non-immunogenic murine iPSC ahead of clinical trials.

## Conclusions

The most exciting aspect in the field of iPSC technology is the clinical application ongoing at RIKEN to treat AMD with RPE cells. AMD is a unique disease to treat with iPSC technology because the RPE cells used are terminally differentiated, and the anatomical site of injection, the eye, is contained and easily recovered in case of any side effects. For other diseases or conditions where systemic cell therapy treatment is needed or the iPSC-derived cells are still capable of proliferation or differentiation, additional regulations will be needed. Cell production issues such as genetic stability with expansion *in vitro*, pluripotent capacity, homogenous populations will demand more in-depth analysis of iPSC than the standard cytogenetic tests currently used. Regulatory issues for clinical trials will have to be developed, including comprehensive SOP for iPSC production, expansion, differentiation and delivery. It will not be feasible to regulate each cell line as a new entity due to time and resource reasons, so there will need to be a dialogue between researchers and regulators to resolve this. There needs to be careful consideration of the type of application for which cells differentiated from iPSC will be used. These issues may not be relevant for grafts that augment or restore function, such as pancreatic beta cell grafts to supplement insulin production or the grafting of cardiomyocytes into a damaged heart. However, the holy grail of accomplishing complete human organ replacement is still a high aspiration target of stem cell biology, and this highlights an additional level of complexity required in the coordinated differentiation and integration of such with pre-existing host tissues. The future looks bright and the whole world awaits the pioneering RIKEN trial for macular degeneration that will point the way for treatment of other diseases or conditions in the near future.

## Abbreviations

ACT: advanced cell technology
ALS: amyotrophic lateral sclerosis
AMD: age-related macular degeneration
CGH: comparative genomic hybridization
FGF: fibroblast growth factor
FISH: fluorescent *in situ* hybridization
GMP: good manufacturing practices
hESC: human embryonic stem cells
hiPSC: human induced pluripotent stem cells
HLA: human leukocyte antigen
iPSC: induced pluripotent stem cells
LIF: leukaemia inhibitory factor
miPSC: mouse induced pluripotent stem cells
NGS: next-generation sequencing
RCS: Royal College of Surgeons
RIKEN: Rikagaku Kenkyujo (Japanese Research Institute)
RPE: retinal pigment epithelial cells
SNPs: single nucleotide polymorphisms
SOP: standard operating procedures
UCL: University College London
VEGF: vascular endothelial growth factor
XIST: transcription X chromosome inactivation
